# The emerging field of opportunities for single-cell DNA methylation studies in hematology and beyond

**DOI:** 10.3389/fmolb.2023.1286716

**Published:** 2023-10-26

**Authors:** Leone Albinati, Agostina Bianchi, Renée Beekman

**Affiliations:** ^1^ Centre for Genomic Regulation (CRG), Barcelona Institute of Science and Technology (BIST), Barcelona, Spain; ^2^ Universitat Pompeu Fabra (UPF), Barcelona, Spain; ^3^ Centre Nacional d’Anàlisi Genòmica (CNAG), Barcelona, Spain; ^4^ Institut d’Investigacions Biomèdiques August Pi i Sunyer (IDIBAPS), Barcelona, Spain

**Keywords:** DNA methylation, single-cell technologies, hematopoiesis, hematological malignancies, epigenomics

## 1 Introduction

**FIGURE 1:**
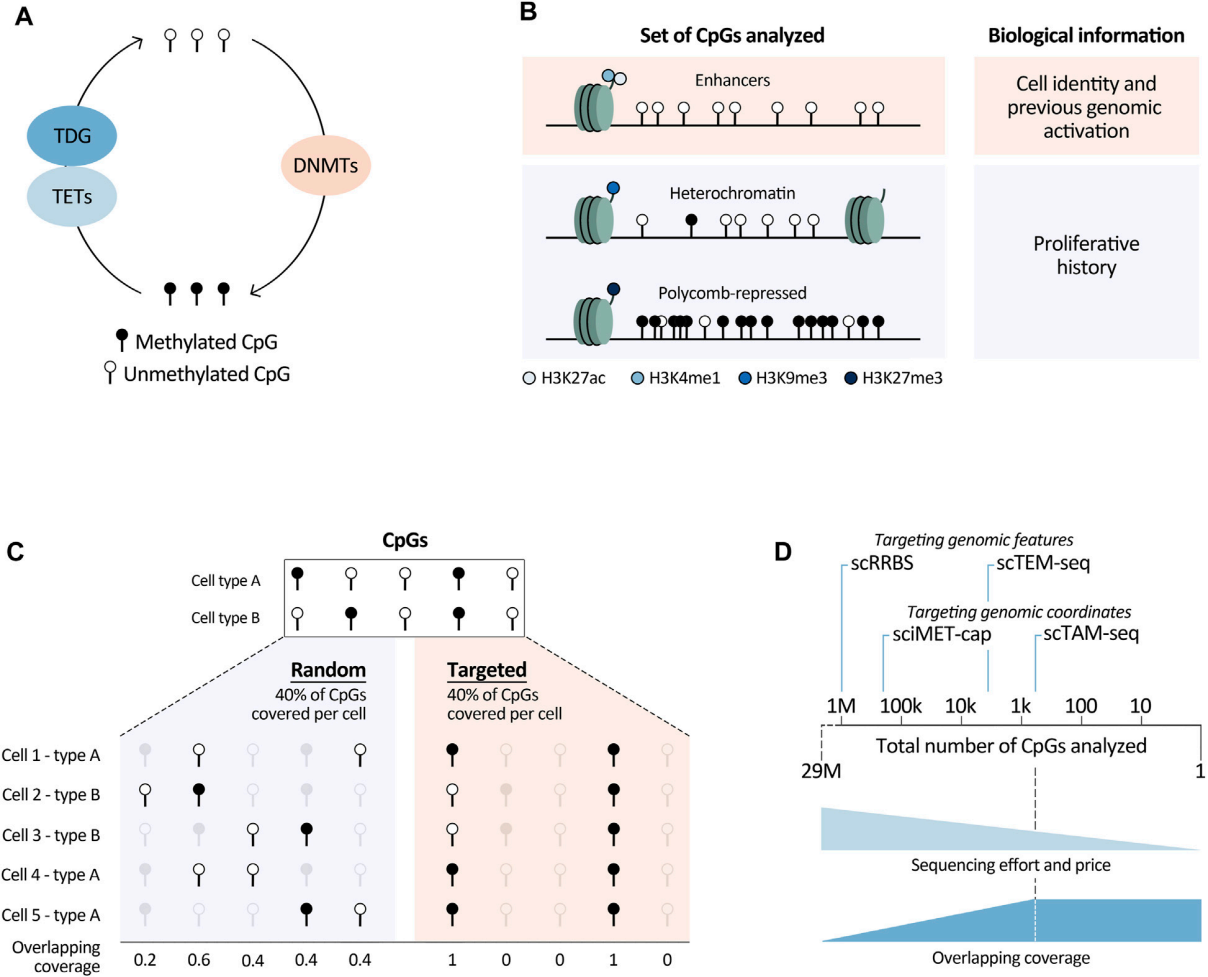
Summary of relevant biological and technological (single-cell) DNA methylation analyses aspects. **(A)** Main enzymes directly involved in DNAm modulation. TETs, Tet Methylcytosine Dioxygenases; TDG, Thymine DNA glycosylase; DNMTs, DNA methyltransferases. Deaminating enzymes such as APOBECs and AID were not included in this overview, as they play a less prominent role in this pathway. **(B)** Different sets of CpGs that can be analysed to retrieve underlying biological information. H3, histone 3; K4/9/27, lysine 4/9/27; me1/3, mono/trimethylation; ac, acetylation. **(C)** Graphical overview of overlapping coverage, indicated by the number of cells in which information of a specific CpG is available. Targeting scDNAm methods to a specific set of the CpGs in the genome increases overlapping coverage (right) in comparison to randomly covering the same percentage of CpGs in each cell (left). **(D)** Targeted methods reduce sequencing efforts and price, while increasing overlapping coverage. They can be based on targeting genomic features such as CpG dense regions in scRRBS and transposable elements in scTEM-seq (for this method the number of CpGs is an approximation based on the number of transposable elements covered per cell), or designed to capture information at specific genomic loci marked by their coordinates (sciMET-cap and scTAM-seq). The latter methods are most powerful to increase overlapping coverage, which we assume reaches a plateau when a low number of CpGs are analysed. This plateau is dependent on the technology and sequencing depth. We believe that this plateau is currently reached at around 900 CpGs, using scTAM-seq. With the speed of technological developments and decrease of sequencing costs this number will increase.

DNA methylation (DNAm) commonly refers to the methylation of cytosines in the mammalian genome, which mainly occurs at CpG sites (cytosines followed by guanines). Its existence was initially predicted in 1948 (Hotchkiss, 1948) and the first analyses of eukaryotic DNAm states were performed in 1978 (Bird and Southern, 1978). Since then, and speeded up by recent genome-wide analyses, a large body of evidence suggests that proper DNAm is vital for hematopoiesis. For example, aberrations affecting the function of the DNAm machinery, e.g., DNA methyltransferases (DNMTs) and demethylating enzymes (TETs among others) (Figure 1A), have profound effects on the hematological system, from differentiation defects to malignant transformation, as reviewed by Gore and Weinstein (2016) and Medeiros et al. (2017). While DNAm is usually associated with gene expression changes, a clear link between these two layers is still lacking. Less commonly considered is that DNAm can be used as read out of other layers of information, such as cell identity (Ziller et al., 2013; Farlik et al., 2016; Loyfer et al., 2023) and cell proliferation (Yang et al., 2016; Youn and Wang, 2018; Zhou et al., 2018; Duran-Ferrer et al., 2020; Minteer et al., 2023). This, together with advancements in single-cell technologies, opens up new avenues to study cellular processes from the single-cell DNAm (scDNAm) perspective. In this opinion piece, we address the main processes that shape the DNA methylome during hematopoiesis and beyond, and provide insights into how to utilise scDNAm studies to extract underlying layers of biological information. Altogether, allowing to expand the opportunities for new discoveries in the single-cell era.

## 2 Leveraging DNA methylation to extract underlying features of normal and tumor cells

Different biological processes shape the DNA methylome of normal and malignant cells. These comprise activation of lineage-specific, cell-stage specific, or tumor-specific enhancers, as well as stochastic errors of DNAm maintenance during cell division, and deposition of DNAm at polycomb-repressed regions. Here we focus on how DNAm changes affected by these different processes can be leveraged to infer other information, such as cell identity, proliferative and genome activation history, and the cell of origin of tumor cells. To understand how the abovementioned features can be extrapolated using this epigenetic mark, DNAm changes targeting regulatory elements and proliferation-associated epigenetic drift will be discussed. We believe that deeper insights into these processes and better knowledge of the CpGs that they target will allow for more comprehensive biological interpretations of large-scale DNAm studies.

DNAm changes in regulatory elements are widely studied since early observations that promoters of tumor suppressor genes (TSGs) are hypermethylated in cancer with p16 as hallmark example (Gonzalez-Zulueta et al., 1995; Herman et al., 1995). However, in many cases silencing of TSGs precedes promoter DNAm and demethylation does not necessarily reactivate gene expression as reviewed by Jones (2012). In addition, promoter DNAm is not directly linked to gene expression, but majorly depends on promoter CpG density (Weber et al., 2007). Nevertheless, for a fraction of promoters, DNAm and gene expression levels show clear correlations, for instance mediated by methylation sensitive transcription factors (TF) (Domcke et al., 2015; Maurano et al., 2015; Grand et al., 2021). Interestingly, with the advancement of genome-wide methods, the epigenomics field has opened up to explore DNAm at enhancers. Even though active enhancers harbour low DNAm levels (Stadler et al., 2011; Ziller et al., 2013), it remains unclear if DNA demethylation is necessary for enhancer activation. For most enhancers chromatin accessibility is not linked to low DNAm levels at the single-molecule level (Kreibich et al., 2023). This suggests that DNA demethylation is not instructive for activation of most enhancers. Nevertheless, enhancer DNAm is a powerful tool to study lineage specification and cell differentiation, both guided by TF programs driving unique enhancer activation patterns. In fact, it is becoming increasingly clear that differential DNAm among cell lineage and differentiation stages are mainly located in enhancers, as shown by detailed DNAm maps of hematopoietic cells (Farlik et al., 2016; Roy et al., 2021), as well as for a broad set of other cell types (Ziller et al., 2013; Loyfer et al., 2023). Enhancer DNAm can furthermore be used as an indirect readout of cellular TF activity, both in healthy and tumor cells. This can be achieved by applying TF motif enrichment analyses in differentially methylated regions, as shown for hematopoeitic cells types and their derived malignancies, as well as for non-hematopoietic cells (Ziller et al., 2013; Kulis et al., 2015; Farlik et al., 2016; Duran-Ferrer et al., 2020; Roy et al., 2021; Loyfer et al., 2023). Finally, genomic regions with low DNAm levels can represent inactive sites (lacking histone marks H3K27ac and H3K4me1) that were active enhancers during earlier cell stages. Clear examples are murine embryonic enhancers with low DNAm levels in adult tissues (Hon et al., 2013; Jadhav et al., 2019). Hence, low DNAm levels at these regions serve as an imprint of past enhancer activity and can be used to study cellular history.

The clear segregation of cell types and cell stages based on DNAm levels at enhancers implies that DNAm profiles can be very informative to determine the cellular origin of tumors. This is of particular interest in poorly differentiated tumors with unclear cell of origin, or in cases of metastasis where the primary tumor cannot be determined. In fact, ample examples exist where DNAm of tumor DNA or circulating cell-free DNA can be used as biomarker to diagnose tumors, as reviewed or analysed in large cohort studies (Liu et al., 2020; Barefoot et al., 2021; Danilova et al., 2022; Stackpole et al., 2022). Moreover, DNAm cannot only be exploited to study tumor cell of origin at the global cell type level, but also at the more detailed level of differentiation stage. For instance, different B-cell related tumors, such as chronic lymphocytic leukemia (CLL) and mantle cell lymphoma, can be further divided into subtypes originating from pre- or post-germinal center B cells (Kulis et al., 2012; Queiros et al., 2016), being relevant for clinical decision making and prognostic outcome.

Intriguingly, DNAm outside enhancers can also be leveraged to understand cellular history. Cell division, for example, is accompanied by global DNAm changes, leaving an epigenetic mark of proliferation. It is one of the main contributors that shape the DNA methylome in tumors as well as healthy cells (Yang et al., 2016; Youn and Wang, 2018; Zhou et al., 2018; Duran-Ferrer et al., 2020; Minteer et al., 2023). This proliferation-associated drift is characterized by stochastic loss of DNAm in heterochromatic regions (lacking the main regulatory histone marks or containing H3K9me3). In contrast, polycomb-repressed regions (marked by H3K27me3) stochastically accumulate DNAm upon proliferation (Duran-Ferrer et al., 2020). Importantly, while this is a relatively novel concept, the underlying observations are not new; global DNAm loss in tumors and DNAm gain at TSG promoters were already observed long ago (Feinberg and Vogelstein, 1983; Gama-Sosa et al., 1983; Gonzalez-Zulueta et al., 1995; Herman et al., 1995), followed by many similar findings. All these likely represent proliferation-associated drift. Of further note, the two contrasting behaviours - loss and gain of DNAm - at different genomic loci usually co-exist within a tumor, but some neoplasms show a clear bias for one of the two (e.g., hypermethylation in acute lymphoblastic leukemia and hypomethylation in multiple myelome) (Duran-Ferrer et al., 2020). Thirdly, while some CpGs targeted by proliferation-associated drift overlap with CpGs affected by ageing, others do not, suggesting that these two processes are related but not necessarily affect the same genomic regions (Duran-Ferrer et al., 2020; Minteer et al., 2023). Though the functional impact of proliferation-associated DNAm drift is still unknown, notion of this process is crucial to investigate cancer development and progression. It can, for example, be used to study the impact of genetic alterations on proliferation or as powerful independent prognostic marker (Duran-Ferrer et al., 2020). In contrast, it also can hamper DNAm interpretations. Particularly, the presence of partially methylated domains due to the stochastic nature of proliferation-associated drift limits the ability to distinguish functional hemi-methylation events, e.g., imprinted or mono-allelic changes, unless moving to the single-cell and/or single-molecule level.

In summary, in this section we have outlined the power of DNAm data to characterise important underlying biological features of normal and tumor cells. All stands or falls though, with selection of the right CpGs. Namely, to study cell identity, CpGs in enhancers marked by histone marks H3K27ac and H3K4me1 will be most powerful. In contrast, for proliferative history, CpGs in H3K9me3-and H3K27me3-marked regions need to be analysed, together with those in heterochromatic regions lacking main regulatory histone marks (Figure 1B). Of note, these regions can differ dependent on the biological system. To define them, detailed, cell-type specific histone-mark based chromatin state maps are needed. In this respect, large-scale efforts to generate reference epigenomes are invaluable (Roadmap Epigenomics et al., 2015; Stunnenberg et al., 2016), with the BLUEPRINT consortium being at the forefront of providing detailed characterisations of normal and malignant hematopoietic cells (Martens and Stunnenberg, 2013). They provide excellent resources enhancing the opportunities to read out underlying biological information from DNAm data.

## 3 Challenges, opportunities, and recommendations for single-cell DNA methylation analyses

As shown in the previous paragraphs, bulk DNAm studies have contributed to discoveries in normal hematological development and its derived malignancies. However, these analyses do not enable the exploration of cellular heterogeneity, nor the detection of rare cell types such as low abundant healthy or pre-malignant cell states, respectively arising during normal differentiation and tumor formation. Neither do they allow us to study DNAm patterns in isolated small cell populations, due to the high amount of input material needed. To overcome these limitations, the DNAm field has directed major efforts towards the development of single-cell technologies. In this section, we will give an overview of the main recent advancements of scDNAm methods and highlight key aspects to take into consideration to choose the appropriate method dependent on the biological question of interest. An overview of the highlighted methods and their main features can be found in Table 1.

**TABLE 1:** Overview of highlighted single-cell DNA methylation methods. Brief overview of the methods mentioned in the text with their main features.

Method	Basis	Platform	Cell throughput*	Average CpG coverage**	Comments	References
DARE	MSRE	Plate based	Low	Genome-wide (400k CpGs/cell, 1.2M total CpGs)	Multiomics allowed: combination with CNV readout	Viswanathan et al. (2019)
Drop-BS	Bisulfite conversion	Droplet based	High	Genome-wide (10k CpGs/cell, 11M total CpGs)	Fast and very high-throughput	Zhang et al. (2023)
epi-gSCAR	MSRE	Plate based	Low	Genome-wide (500k CpGs/cell)	Multiomics allowed: combination with SNV readout	Niemöller et al. (2021)
sciMET/ sciMETv2	Bisulfite conversion	Plate based	High	Genome-wide (2M CpGs/cell, 12M total CpGs)	High-throughput plate-based method using combinatorial indexing	Mulqueen et al. (2018) Nichols et al. (2022)
sciMET-cap	Bisulfite conversion	Plate based	High	Targeted to regulatory elements (200k CpGs/cell)	High-throughput plate-based method using combinatorial indexing and capture probes	Acharya et al. (2023)
scRRBS	Bisulfite conversion	Plate based	Low	Targeted to promoters (250k—1M CpGs/cell)	Widely developed and utilized method. Newer versions allow multiomics: combination with whole transcriptome and/or somatic mutations readouts	Guo et al. (2013) Gaiti et al. (2019) Gu et al. (2021)
scTAM-seq	MSRE	Droplet based	High	Tageted to customized CpGs of interest (400–900 CpGs/cell)	Multiomics allowed: combination with somatic mutations, and cell-surface markers readouts	Bianchi et al. (2022)
scTEM-seq	Bisulfite conversion	Plate based	Low	Targeted to SINE Alu and LINE-1 transposable elements	Multiomics allowed: combination with whole transcriptome readout	Hunt et al. (2022)
snmC-seq/ snmC-seq2	Bisulfite conversion	Plate based	Low	Genome-wide (1.5M CpGs/cell)	Even though this is a low-throughput method, in Luo *et al.* (2017) DNAm profiles of > 1,000 cells were generated	Luo et al. (2017) Luo et al. (2018)

MSRE, methylation-sensitive restriction enzymes; CNV, copy number variation; SNV, single nucleotides variant. *We considered as high-throughput methods those that easily allow to reach an output of >1,000 cells per sample. **We separated methods based on being targeted or genome-wide and we highlighted the number of CpGs per cell when clearly stated in the corresponding papers.

Single-cell DNAm methods face multiple challenges, both from the biological and technical perspective. A biological bottleneck is the large number of CpGs present in every human cell, namely, two copies of roughly 29M CpGs. Therefore, analyzing the entire DNA methylome in large numbers of cells is very difficult, if not impossible, due to sequencing limitations. Covering all CpGs in a single cell would require 100–1,000 times more reads/cell in comparison with a scRNA-seq experiment. Even with extreme sequencing efforts (5–7M reads/cell), genome-wide single-cell methods can only cover a random set of 6%–7.5% of the total number of CpGs/cell (Luo et al., 2017; Mulqueen et al., 2018). Consequently, scDNAm methods suffer from high dropout rates, generating very sparse datasets. This sparsity is furthermore augmented by other technical reasons such as degradation of the DNA upon bisulfite treatment and PCR amplification biases or failures. Importantly, the high level of randomness of CpG coverage in genome-wide methods implies that it is unlikely to have the same CpGs covered in different cells, resulting in a low so-called overlapping coverage. In other words, for each cell one obtains information about a different set of CpGs (Figure 1C). This leads to the loss of single-CpG resolution when comparing DNAm readouts among cells, since binarization of the data into larger regions is necessary. A related biological limitation is that most CpGs have static DNAm levels; within each tissue only a minor fraction of CpGs show dynamic methylation (Kulis et al., 2015; Durek et al., 2016; Loyfer et al., 2023). This means that only a small fraction of the DNA methylome is informative. For the above-mentioned reasons, aiming to analyze the entire DNA methylome, as in genome-wide methods, hinders the ability to obtain information about biologically relevant CpGs.

Nevertheless, genome-wide bisulfite-based scDNAm methods, such as sci-MET (Mulqueen et al., 2018), sci-METv2 (Nichols et al., 2022), snmC-seq (Luo et al., 2017), snmC-seq2 (Luo et al., 2018) and Drop-BS (Zhang et al., 2023) offer exciting opportunities to perform hypothesis-free DNAm analyses, with high-throughput methods enabling readouts of thousands of cells. However, one must keep in mind that the overlapping coverage and resolution obtained with these methods largely depend on the sequencing depth one can afford. At high sequencing depth they are effective in distinguishing cell types. Hence, taking advantage of their unbiasedness, genome-wide methods have been used to build cell-type specific DNAm atlases (Luo et al., 2017), investigate hematopoiesis during embryonic development (Mukamel et al., 2022), and study hematopoietic stem cell populations (Hui et al., 2018). This illustrates their power to uncover new cell types and intermediate differentiation stages. Additionally, even with low sequencing depth we consider that they can be employed to study proliferation-associated drift, as this can be calculated using DNAm levels over large genomic regions. While sequencing depth is of major importance, increased sequencing efforts cannot control for the loss of information due to degradation of DNA during bisulfite conversion. For this reason, alternative genome-wide methods based on methylation-sensitive restriction enzymes (MSRE) have been developed, such as epi-gSCAR (Niemoller et al., 2021) and DARE (Viswanathan et al., 2019). MSRE-based methods improve overlapping coverage, but at the same time they are not completely unbiased, since CpGs should be located inside an MSRE restriction site to be studied. Furthermore, they are plate-based, which limits cellular throughput to a few cells per experiment, unless one can count on the use of a robot to automate the plate-based processing.

To overcome constraints related to the analysis of the entire DNA methylome, we and others have developed targeted scDNAm methods. We saw the opportunity of combining MSRE with a microfluidics system, the Tapestri platform from Mission Bio, and developed scTAM-seq (Bianchi et al., 2022). Our methodology builds upon an important development to reduce the needed sequencing depth (and thus the price) by targeting biological informative CpGs (Figure 1D). Other targeted methods have so far focused on covering specific functional regions in the genome such as regulatory regions in sciMET-cap (Acharya et al., 2023), promoters in scRRBS (Guo et al., 2013; Gaiti et al., 2019; Gu et al., 2021), or LINEs and SINEs in scTEM-seq (Hunt et al., 2022). While these methods do reduce sequencing requirements, they still suffer from low overlapping coverage. Furthermore, scRRBS and scTEM-seq are plate-based which limits cellular throughput as mentioned above. Yet, such approaches have shed important light onto DNAm dynamics in relation to clonal hematopoiesis and CLL (Gaiti et al., 2019; Izzo et al., 2020). To maximize overlapping coverage and allow cell comparisons of a highly informative set of methylation sites at the single-CpG level, we developed the microfluidics-based method scTAM-seq, using a rigorous selection of approximately 400 genomic sites. This method allowed us not only to read out distinct B-cell stages but also to capture the continuous process of memory B-cell formation (Bianchi et al., 2022). Of notice, this method requires prior knowledge of the system to design the CpG panel. Nevertheless, we believe that our method will open new doors to characterise complex, continuous biological systems with transitional cell states such as hematological differentiation (Velten et al., 2017; Weinreb et al., 2020), cellular reprogramming (Kong et al., 2022), as well as tumor development and plasticity (Kinker et al., 2020; Nadeu et al., 2022).

Overall, in this section, we have discussed the major aspects to consider when opting to use scDNAm methods. The most suitable method to profile the DNA methylome in single cells will depend on 1) the knowledge of the model, 2) the biological question to be answered, and 3) the budget. That being said, to obtain a global snapshot of the DNAm landscape or build cellular DNAm atlases, unbiased genome-wide methods are the most appropriate (Luo et al., 2018; Nichols et al., 2022; Zhang et al., 2023), followed by less-expensive targeted approaches such as scRRBS (Guo et al., 2013) or sciMET-cap (Acharya et al., 2023). Based on the number of input cells, either plate-based (low input) or droplet-based/combinatorial-indexing-based (high input) methods would be preferred. Finally, if one aims to study a small set of highly informative CpGs (up to 900) at single-CpG level, we believe that scTAM-seq is optimal (Bianchi et al., 2022). The overlapping coverage that can be achieved by this method allows the study of DNAm variability at individual genomic loci as well as their combinatorial DNAm behaviors in unprecedented detail.

## 4 Discussion

The single-cell field has evolved extensively in recent years, providing highly informative atlases of many tissue types through large-scale initiatives such as the Human Cell Atlas (Regev et al., 2017). The field has largely focused on transcriptomic and chromatin accessibility readouts though, with single-cell explorations of the DNA methylome lagging far behind. This implies that our view of the DNA methylome at single-cell level is still in its infancy, while highly relevant information can be extracted from this epigenetic layer. In this opinion piece, we describe the underlying biological features that can be read out from DNAm data and we lined out the main developments of the scDNAm field in recent years. In this way, we aim to underline the biological relevance of DNAm studies in the single-cell era, an emerging field with many opportunities. Importantly, the scDNAm field is rapidly progressing and while we write this piece, new technologies are being developed with the aim to increase the targeted amount of CpGs, overlapping coverage, and resolution, while reducing sequencing efforts and therefore the costs. Furthermore, though out of the scope of this piece, techniques are developed to combine DNAm studies with other (epi)genetic layers of information (Viswanathan et al., 2019; Niemoller et al., 2021; Bianchi et al., 2022; Angermueller et al., 2016; Hou et al., 2016; Guo et al., 2017; Clark et al., 2018; Linker et al., 2019; Gu et al., 2021; Hunt et al., 2022). Such single-cell multi-omics technologies shall aid in better understanding of the correlations between DNAm and other molecular features such as somatic mutations, copy number variations, chromatin accessibility, gene expression, cell-surface proteins, and alternative splicing. In summary, with all recent biological insights and technical advancements, the scDNAm field has exciting times ahead, heading towards deep characterisations of complex biological processes such as cell differentiation, cellular reprogramming, and tumor formation, capturing the potential paths cells can follow within these continuous landscapes.
